# Circulating microRNA-92a level predicts acute coronary syndrome in diabetic patients with coronary heart disease

**DOI:** 10.1186/s12944-019-0964-0

**Published:** 2019-01-22

**Authors:** Wenyi Wang, Zhigang Li, Yashu Zheng, Meiling Yan, Yameng Cui, Jiechun Jiang

**Affiliations:** 1 International Medical Center, Tianjin First Central Hospital, No. 24 of Fukang Road, Nankai District, Tianjin, 300192 China; 2 Pharmacy Department, Tianjin First Central Hospital, Tianjin, China; 3 Medical Laboratory, Tianjin First Central Hospital, Tianjin, China

**Keywords:** Acute coronary syndrome, microRNA-92a, Type 2 diabetes mellitus, Coronary heart disease

## Abstract

**Purpose:**

This study was designed to explore the value of monitoring miR-92a in T2DM patients with coronary heart disease (CHD).

**Materials and methods:**

40 ACS patients with prior history of CHD and diabetes while the onset time of diabetes preceded that of CHD by more than 2 years were enrolled as the DACS group(diabetic ACS group). 40 ACS subjects who had had a definite diagnosis of CHD for more than 2 years with no history of T2DM were recuited as the CACS group(chronic CHD with ACS group). All enrolled subjects from DACS and CACS group came from an emergency basis and diagnosed with ACS by coronary angiography. Another 68 age- and sex-matched volunteers with chronic stable CHD without diabetes history were assigned as the control group (CHD group). We examined the serum levels of miR-92a and analyzed their correlations with blood pressure, glucose level, and lipid level.

**Results:**

The levels of miR-92a were significantly elevated in the DACS group compared with those of the CACS and CHD groups. Multivariate analysis showed that miR-92a, systolic blood pressure (SBP), and glycosylated hemoglobin (HbA1c) were significantly related to ACS events in patients with T2DM. Forward stepwise binary logistic regression analysis identified miR-92a as an independent predictive factor for ACS events in the patients with T2DM.

**Conclusion:**

An elevated circulating miR-92a level was associated with an increased risk of ACS in CHD patients with T2DM. Thus the level of miR-92a, especially combined with elevated SBP and HbA1c, may be helpful in the detection of ACS in patients with T2DM.

**Electronic supplementary material:**

The online version of this article (10.1186/s12944-019-0964-0) contains supplementary material, which is available to authorized users.

## Introduction

Acute coronary syndrome (ACS) is an acute cardiac ischemic syndrome due to coronary artery atherosclerotic thrombosis involving the rupture or erosion of unstable plaques [1]. More than half of cardiovascular deaths are caused by ACS, and hyperglycemia is an independent risk factor for cardiovascular events [2]. In short, patients suffering from type 2 diabetes mellitus (T2DM) and ACS face an increased risk of adverse cardiovascular events.

Patients with coronary heart disease (CHD) and T2DM, as compared with nondiabetic patients, often have complicated vascular lesions of increasing severity as well as multivessel and diffuse vasculopathy [3]. Previous studies point to several circulating biomarkers related to the incidence of ACS in T2DM patients, such as glycosylated hemoglobin (HbA1c) [4], B-type natriuretic peptide (BNP) [5], phospholipid protein [6], gremlin 1, and macrophage migration inhibitory factor [7]. However, these biomarkers are of limited use because they are nonspecific. Therefore the clinical diagnosis of blood glucose–related ACS events still focuses on the typical clinical symptoms and findings of invasive imaging. Because of this, it would be useful to find specific markers that could facilitate early intervention in ACS patients with T2DM. In other words, this would provide a new approach of great socioeconomic value for the prevention and treatment of vascular complications in patients at risk.

As small noncoding RNA molecules, the microRNAs (miRNAs) regulating gene expression at the posttranscriptional level have emerged as playing a fundamental role in many diseases. In biological research much has been achieved regarding the miRNAs, which are now known to participate in the pathogenesis of T2DM mellitus and related complications [8]. Accordingly, studies have found aberrant expression of miR-143, miR-21, miR-486-3p, miR-100, miR-92a, miR-3135b, and miR-223 in ACS patients [9–15]. In studies of ACS in T2DM (referred to here as diabetic ACS, or DACS), miR-92a attracts particular attention because it is a newly recognized biomarker of ACS [13] and hyperglycemia [16] in patients with CHD; also, it regulates neovascularization, and the inhibition of miR-92a enhances angiogenesis [17]. There is compelling evidence that miR-92a plays a role in the pathogenesis of ACS and that high levels of expression of miR-92a can serve as a potential biomarker to differentiate between patients with stable CAD and those with acute myocardial infarction (AMI). In AMI, miR-92a can even point to the activation of endothelial cells [18]. In coronary sinus samples, miR-92a-3p was found to be significantly downregulated, and left ventricular filling pressure was negatively correlated with miR-92a-3p [19]. How miRNA-92a exerts its influence on the pathogenesis of T2DM and related complications, especially ACS in CHD, is unclear. We tested the hypothesis that levels of miR-92a would have a significant positive correlation with rates of morbidity and mortality in CHD patients with T2DM and that levels of miR-92a could be assessed from samples of peripheral blood. In order to test this hypothesis, we studied both the expression pattern and clinical relevance of circulating miR-92a with T2DM in CHD and ACS patients with T2DM.

## Material and methods

### Ethics

This study was approved by the Ethics Committee of Tianjin First Center Hospital in Tianjin, China. Each participant in the study received both written and oral information concerning the goals of the study and each gave informed consent.

### Patients

Inpatients with ACS from October 2014 to March 2016 were recruited in this study. According to current guidelines on ACS, eligible ACS subjects included patients with symptoms of decreased blood flow to the heart, such as chest pains, ST-segment elevation on the ECG, abnormal myocardial markers such as cardiac troponin (cTn), and/or a D-dimer. We found more than 230 patients who had been hospitalized on an emergency basis and, after coronary angiography, had been diagnosed with ACS. T2DM history, random blood glucose (RBG), HbA1c, fibrinogen (FIB), cTn, and blood lipids were recorded immediately on admission. After diabetic microangiopathy screening—including temperature sensory examination, nylon inspection, vibration examination, and electroencephalography—T2DM subjects with microvascular complications were excluded. The DACS group included those ACS patients who had had a history of T2DM for more than 2 years and the onset time of diabetes preceded that of CHD by more than 2 years. The CACS group included those ACS patients who had had a definite diagnosis of CHD for more than 2 years with no history of T2DM. Finally, 40 cases were enrolled as the DACS group and another 40 with no diabetes history as the CACS group. Another 68 age- and sex-matched volunteers with chronic stable CHD were recruited to serve as the control group (CHD group). Chronic stable coronary heart disease includes stable angina pectoris diagnosed by the load ECG exercise test and coronary artery angiography.

The following conditions were excluded: (1) type 1 diabetes mellitus (T1DM), diabetic ketoacidosis, and coma; (2) malignant tumor, diseases of the hematologic system, immune system diseases; (3) acute inflammation, hemorrhage, pregnancy; (4) severe hepatic and/or renal dysfunction, psychological problems.

### Clinical measurements

An Omron HEM-7136 monitor (Japan) was adopted to measure diastolic blood pressure (DBP) and systolic blood pressure (SBP). Chemiluminescence immunoassays were used to determine the plasma cTn concentration according to the protocol of the manufacturer (Beckman Coulter, Fullerton, CA). Plasma random blood glucose (RBG), high-density lipoprotein cholesterol (HDL-C), low-density lipoprotein cholesterol (LDL-C), total glyceride (TG), and total cholesterol (TC) levels were all determined by an enzymatic method using reagents from Roche Diagnostic on a Roche Automatic Biochemical Analyzer Modular DPP (Roche, Switzerland). FIB levels were determined using a coagulation method on an ACL-TOP700 instrument (Walfen, USA). HbA1c was detected by ion-exchange high-performance liquid chromatography on a Bio-Rad Variant™II Hemoglobin Testing System (BIO-RAD, USA). All results were carried out as means of 3 tests.

### Processing of samples and RNA extraction

Peripheral blood (2 mL) from each participant was placed in an heparin tube. A commercial Trizol reagent (Invitrogen, Carlsbad, CA,) was utilized to isolate the total RNA from serum according to the protocol of the manufacturer. Samples were then lysed in Trizol reagent and mixed with chloroform. The lysate was centrifuged so that protein, DNA, and RNA could be separated; the serum-Trizol homogenate wAS centrifuged at 12,000 r/min for 10 min at 4 °C; the supernatant was then transferred to microcentrifuge tubes. Total RNA was recovered with the precipitation of isopropanol and rinsed in 75% ethanol to eliminate impurities. Finally the total RNA was dissolved in RNase-free water and concentrated in a volume of 20 μL with an Eppendorf Concentrator Plus 5301 (Eppendorf, Germany). A Nano Drop ND-1000 (Nano Drop, Wilmington, DE) was used to quantify the concentration of each RNA sample. All RNA samples were stored at − 80 °C until use.

*miRNA expression and real-time reverse transcriptase polymerase chain reaction (RT-PCR).*

The miRNA was quantified by quantitative and real-time polymerase chain reaction (RT-PCR) from total RNA; it was polyadenylated and reversely transcribed into cDNA in a final volume of 20 μL using the PrimeScript RT reagent kit (Takara, Bio Inc., Tokyo, Japan). RT-PCR was implemented in duplicate measurements by utilizing SYBR Premix Ex Taq™ II (Takara, Bio Inc., Tokyo, Japan) in a 7300 quantitative PCR system (Applied Biosystems). The miRNA-specific primer sequences were designed on the basis of miRNA sequences that were acquired via the miRBase database (http://www.mirbase.org//) (UAUUGCACUUGUCCCGGCCUGU). All amplification reactions were made in one final volume of 20 μL that contained 1 μL of cDNA, 0.25 mm of primer, and 1× SYBR Green PCR Master mix in accordance with the cycling conditions recommended by the supplier (40 cycles of 30 s at 90 °C, 5 s at 95 °C, and 31 s at 60 °C). Upon completion of the PCR cycles, melting curve analyses and electrophoresis of products on 3.0% agarose gels were conducted to corroborate generation of the anticipated PCR product. All samples were run in triplicate. The levels of expression of miRNAs were normalized to miR-92a, and calculated using the 2^-ΔΔct^ method.

### Statistical analysis

SAS software, version 9.3 (SAS Institute, Cary, NC), was used to conduct statistical analyses. Continuous variables were demonstrated as averages (standard deviation) and compared by one-way analysis of variance (ANOVA) or median (interquartile range) and Kruskal-Wallis analysis according to the Shapiro-Wilk test. These analyses were followed by Bonferroni comparisons. Each probability value was two-sided and a *P* value < 0.05 was considered statistically significant. The correlation between miR-92a level and clinical data—including hemodynamics parameters, blood glucose parameters, and blood lipid parameters—were all evaluated by the calculating Spearman’s rank correlation coefficients. The curve analysis of receiver operating characteristics (ROCs) was used to assess the diagnostic accuracy of serum biomarker levels. The optimal cutoff values for defining DACS were calculated on the basis of maximizing the sensitivity and specificity of each index. Analyses of forward stepwise binary logistic regression were conducted to determine the optimal combination of biomarkers so that DACS could be predicted.

## Results

### Patient characteristics

A total of 68 CHD control subjects, 40 CACS patients, and 40 DACS patients were included in this study. The participants’ mean age and sex were similar.

The mean RBG was 4.86 mmol/L (interquartile range [IQR] 4.42–5.37 mmol/L) in the CHD controls, 9.02 mmol/L (IQR 6.58–12.16 mmol/L) in the CACS group, and 11.51 mmol/L (IQR 8.30–14.39 mmol/L) in the DACS group. The HbA1c was 4.68% (IQR 4.47–4.94%) in the CHD controls, 5.35% (IQR,4.78–5.86%) in the CACS group, and 8.00% (IQR 7.27–9.60%) in the DACS group. Both RBG and HbA1c were dramatically increased in the CACS and DACS groups compared with the CHD control group (*P* < 0.001). The CACS and DACS groups had higher levels of triglyceride (TG) and total cholesterol (TC) than the CHD control group (*P* < 0.001). Both HDL-C and LDL-C were dramatically decreased in the DACS group compared with the CACS group, with 88.57% of the patients in the CACS group having normal levels of statins compared with 95.73% in the DACS group (Table 1).

**Table 1:** Clinical characteristics of enrolled subjects

Variables	CHD controls (n = 68)	CACS (n = 40)	DACS (n = 40)	*P* value
Age, year	47 (44, 52)	52.5 (49.5, 57)	52.5 (50, 57)	0.964
Sex, male %	47 (69.12)	24 (60.00)	32 (80.00)	0.150
BMI, kg/m^2^	20.68 (19.90, 21.47)	20.23 (19.35, 21.12)	20.96 (19.92, 21.66)	0.046
Smoking Index	410 (215, 565)	420 (210, 790)	460 (200, 740)	0.645
Hemodynamics				
SBP, mmHg	112 (102.5, 119)	123 (117, 131)^**^	134.5 (124, 154.5)^**, †^	<0.001
DBP, mmHg	74 (69, 81)	79.5 (71.5, 84)	80 (75, 90)^*^	0.003
Coagulation Function				
FIB, g/L	2.55 (2.34, 2.91)	2.81 (2.53, 3.07)	3.18 (2.69, 3.67)^**^	<0.001
D-dimer,µg/L	228.210 (120.86, 303.89)	339.20 (224.65, 491.81)^**^	333.40 (265.21, 417.22)	<0.001
Myocardial Marker				
cTn, ng/ml	0.03 (0.02, 0.04)	0.02 (0.01, 0.03)	0.05 (0.01, 3.07) †	0.010
Blood Glucose				
RBG, mmol/L	4.86 (4.42, 5.37)	9.02 (6.58, 12.16)^**^	11.51 (8.30, 14.39)^**, †^	<0.001
HbA1C, %	4.68 (4.47, 4.94)	5.35 (4.78, 5.86)^**^	8.00 (7.27, 9.60)^**, ††^	<0.001
Blood Lipids				
LDL-C, mmol/L	2.66 (2.42, 2.89)	3.11 (2.63, 3.92)^**^	2.61 (2.15, 3.39)†	<0.001
TG, mmol/L	1.04 (0.77, 1.34)	1.48 (0.85, 2.37) ^**^	1.59 (0.95, 3.40) ^**^	<0.001
TC, mmol/L	3.98 (2.04, 4.76)	4.84 (4.26, 5.42)^**^	4.56 (3.26, 6.27)^*^	<0.001
HDL-C, mmol/L	0.70 (0.41, 1.01)	1.04 (0.87, 1.32)^**^	0.92 (0.55, 1.19)^†^	<0.001
Biomarkers				
miR-92a^a^	2.39 (1.03)	3.88 (0.78)^**^	5.60 (1.22)^**,††^	<0.001
Treatment	antiplatelet therapy (81.16%), nitrates (43.49%), ACEI/ARB (75.36%), statins (79.71%), beta-blockers (40.58%)	antiplatelet therapy (90.00%), nitrates (85.71%), ACEI/ARB (72.86%), statins (88.57%), beta-blockers (54.29%), diuretics (62.86%)	antiplatelet therapy (81.20%), nitrates (70.09%), ACEI/ARB (87.18%), statins (95.73%), beta-blockers (29.91%), biguanides (24.79%), insulins (41.88%)	

#All the continuous variables were compared using Kruskal-Wallis analysis and presented by median (interquartile range) for their non-normal distribution unless there are some additional notes such as a. One-way analysis of variance was adopted to evaluate the equality of marked indicator, which was presented by average (standard deviation). *P < 0.017, **P < 0.001 vs. NC group; †*P* < 0.017, ††*P* < 0.001 vs. CACS group.

**Fig. 1:**
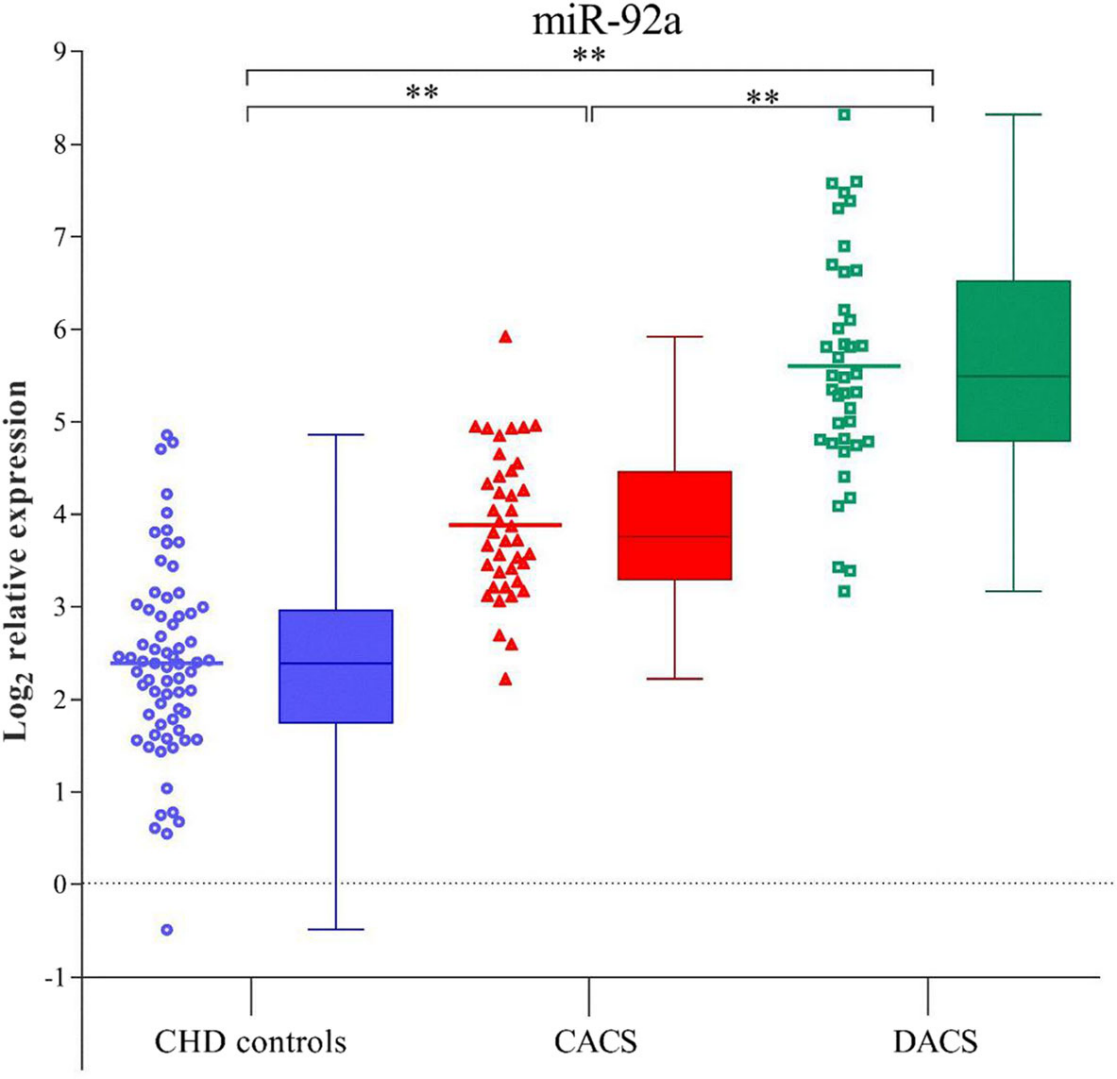
Levels of miR-92a in the CHD, CACS, and DACS groups. The box-and-whiskers plot was done via Turkey method. **p<0.001

Expression of miR-92a was more clearly elevated in the DACS group than that in the CACS and CHD groups (all *P* < 0.017). In addition, an apparent difference was also found between the CACS and CHD groups (Table 1 and Fig. 1).

### Expression of miR-92a

By and large plasma levels of miR-92a were similar in male and female subjects. No correlation was found between age and the level of miR-92a after adjustment by group. However, miR-92a expression was more clearly elevated in the DACS group than in the CACS and CHD groups (*P* < 0.001). In addition, an apparent difference was also found between CACS group and CHD group (Fig. 1, Table 1).

### Correlation between miR-92a level and clinical measurements

The correlation between clinical measurements and miR-92a level was further analyzed. By utilizing Spearman’s rank correlation analysis, it was demonstrated that miR-92a level was correlated with D-dimer after adjustment by group, which is excluded subsequently (Table 2, Additional file 1 Figures S1–S3).

**Table 2:** Spearman`s correlation coefficients of miR-92a level with clinical data

Variables	No adjusted		Adjusted by group	
	*r* value	*P* value	*r* value	*P* value
Age	0.06	0.592	-0.01	0.913
Male Sex	-0.03	0.746	0.05	0.556
BMI	-0.10	0.228	-0.11	0.188
Smoking Index	0.01	0.948	-0.09	0.303
Hemodynamics				
SBP	0.53	<.001	0.09	0.280
DBP	0.26	0.002	0.06	0.454
Coagulation function				
FIB	0.30	<.001	-0.01	0.922
D-dimer	0.17	0.040	-0.21	0.011
Myocardial markers				
cTn	0.15	0.070	0.07	0.379
Blood Glucose				
RBG	0.64	<.001	0.07	0.368
HbA1C	0.60	<.001	0.01	0.909
Blood Lipids				
LDL-C	0.05	0.570	-0.01	0.857
TG	0.22	0.008	-0.15	0.076
TC	0.20	0.013	-0.05	0.517
HDL-C	0.19	0.020	0.06	0.457

### The potential of miR-92a to predict ACS in patients with T2DM

Both univariate and multivariate analyses were further conducted, so that factors associated with the prediction of DACS could be identified. Univariate analysis was adopted to select predictive factors of DACS, revealed that sex, body mass index (BMI), SBP, FIB, cTn, RBG, HbA1c, LDL-C, HDL-C, and miR-92a were all closely associated with DACS. Predictive factors selected from the univariate analysis were utilized as variables for forward stepwise multivariate logistic regression. SBP, miR-92a, and HbA1C were further added to the multivariate logistic regression analysis. miR-92a (OR 95% CI: 11.011 [1.967, 61.630], *P* = 0.006) was confirmed as an independent predictive factor for DACS among CHD patients (Table 3).

**Table 3:** Univariate and multivariate logistic regression analysis of variables related to diabetics ACS due to coronary hear desease

Variables	Univariate analysis		Multivariate analysis	
	*OR (95% CI)*	*P* value	*OR (95% CI)*	*P* value
Age, year	1.001(0.919-1.09)	0.983	—	—
Sex,male %	0.375(0.138-1.020)	0.055	—	—
BMI, kg/m^2^	1.534(0.998-2.357)	0.051	—	
Smoking Index	1.000(0.999-1.001)	0.835		
SBP, mmHg	1.039(1.011-1.067)	0.006	1.127(1.024-1.239)	0.014
DBP, mmHg	1.020(0.977-1.064)	0.366	—	—
FIB, g/L	2.059(1.082-3.919)	0.028	—	—
D-dimer	0.999(0.996-1.002)	0.627		
cTn#, ng/ml	1.824(1.213-2.743)	0.004	—	—
RBG, mmol/L	1.181(1.043-1.336)	0.009	—	—
HbA1C,%	6.147(2.657-14.219)	<.001	25.707(2.279-313.031)	0.009
LDL-C,mmol/L	0.492(0.286-0.846)	0.010	—	—
TG,mmol/L	1.100(0.887-1.365)	0.385	—	—
TC,mmol/L	0.870(0.650-1.164)	0.347	—	—
HDL-C,mmol/L	0.113(0.027-0.485)	0.003	—	—
miR-92a	6.006(2.757-13.085)	<.001	11.011(1.967-61.630)	0.006

Note: #The data was "transformed" by log-exponential transform before being added to the logistic regression

ROC analysis determined the diagnostic accuracy of HbA1C, miR-92a, and SBP, as shown in Table 4. The miR-92a separately (cutoff > 4.65) showed 85% sensitivity and 82.50% specificity with an AUC of 0.883 in the prediction of DACS. The forward stepwise binary logistic regression analysis showed that the combination of HbA1C, SBP, and miR-92a had a better predictive value, with an AUC of 0.986, than the combination of any individual biomarker taken separately.

$$ P=\frac{1}{1+\mathit{\exp}\left(\hbox{-} Z\right)} $$

**Table 4:** Data from ROC curves

Indicators	AUC(95%*CI*)	*P* value	Cut-off	Sensitivity/Specificity	PPV/NPV
SBP	0.687 (0.573,0.786)	0.002	>132	60.00/77.50	72.70/66.00
HbA1C	0.926 (0.849,0.974)	<.001	>6.43	82.50/100.00	100.00/85.10
miR-92a	0.883 (0.791,0.944)	<.001	>4.65	85.00/82.50	82.90/84.60
SBP,HbAIC,&miR-92a^a^	0.986 (0.930,0.998)	<.001	_	95.00/95.00	95.00/95.00

Note: ^a^Multivariate logistic regression analysis is adopted to generate predicted probability; AUC refers to the area under ROC curve; CI is the confidence interval; PPV represents the positive predictive value; NPV is the negative predictive value

The probability of HbA1C, SBP, and miR-92a was calculated by adopting the following logistic formula in the prediction of diabetic ACS: Z = β0 + β1X1 + β2X2+ … + βmXm. β0 is a constant, and β1, β2 … βm refers to the estimated regression coefficients of risk factors. Therefore the predicted probability (*P*) was calculated by the following formula:

$$ P=\frac{1}{1+\exp \left(47.00\hbox{-} {0.12}^{\ast}\kern0.24em SBP\hbox{-} {3.28}^{\ast }\  HbA 1C\hbox{-} {2.40}^{\ast }\  miR\hbox{-} 92a\right)} $$

**Fig. 2:**
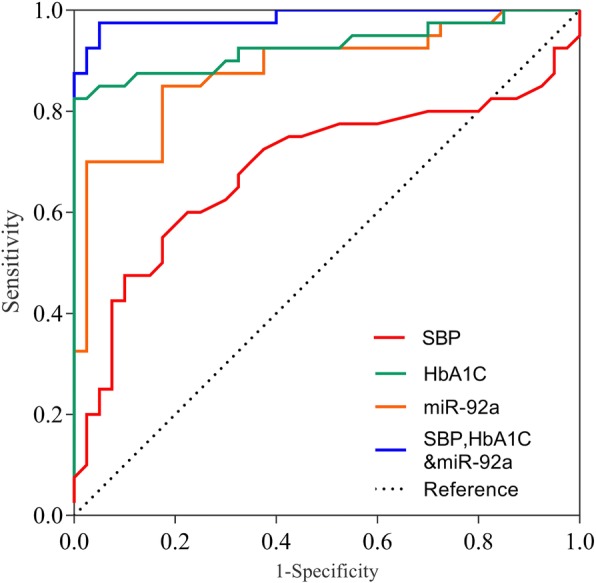
ROC curve analysis. Pairwise comparison of ROC curves: *P* (SBP vs HbA1C) < 0.001; *P* (SBP vs miR-92a) = 0.003; *P* (HbA1C vs miR-92a) = 0.342.

The combination of HbA1C, elevated SBP, and miR-92a demonstrated 95% sensitivity and 95% specificity in predicting DACS among patients with CHD (Table 4, Fig. 2, Additional file 1 Figure S4).

## Discussion

The significance of circulating biomarkers for predicting DACS in CHD patients has aroused great interest. This study demonstrates that miR-92a levels rose dramatically in DACS patients and had no correlation with blood glucose or blood lipids levels. Our univariate logistic regression analysis showed that the miR-92a and SBP, FIB, cTn, RBG, HbA1c, LDL-C, and HDL-C were significantly related to the DACS event. It further demonstrated that miR-92a was an independent predictive factor for the DACS.

The overall incidence of CHD and ACS has been rising, with serious implications for public health; moreover, T2DM is an important risk factor for cardiovascular disease [20]. According to current diabetes guidelines, DACS is a macrovascular complication of T2DM [21]. Compared with the nondiabetic CHD population, the age of onset of atherosclerosis in patients with CHD and T2DM is earlier; its progression is more rapid and complicated by vascular complications, multivessel disease, and other diffuse lesions [22]. The mechanism accelerating atherosclerosis can simultaneously cause an ACS event. Among the specific mechanisms controlling DACS and the integrative function of cardiovascular diseases, noncoding miRNA sequences have drawn much attention [14]. For example, miR-92a is part of the miR-17-92 cluster of miRNAs and has been recognized as a significant proatherogenic mechano-miRNA [23]. miR-92a is expressed in endothelial cells in the aortic arch, and a study by de Winther [24] showed that it induces endothelial dysfunction and a proatherogenic response. Further research showed that miR-92a induced endothelial inflammation by targeting KLF2 and KLF4 [25] or via the NRF2-KEAP1-ARE pathway [26]^,^ even controlling cholesterol levels and Golgi volume via protein secretion [27]. Nevertheless, there is as yet no study showing whether miR-92a plays a role in DACS.

miRNAs can be released into the circulation; they participate in regulating gene expression both intracellularly and at a distance. In our study, 80 patients with ACS were enrolled well as 68 CHD control subjects and their plasma miR-92a levels were analyzed. It was observed that miR-92a level was comparable between male subjects and female subjects. Furthermore, miR-92a levels had no correlation with age or BMI. Nevertheless the circulating level of miR-92a was apparently different between DACS patients and those without diabetes. In particular, miR-92a levels in DACS patients increased to a greater degree than those in nondiabetic ACS patients, implying that miR-92a may play a role in the progression of disease in DACS patients.

In clinical practice, hypertension is regarded as a major risk factor for ACS among CHD patients. Other risk factors include hyperlipidemia, especially TC and LDL-C, hyperglycemia, smoking, obesity, and genetics. In accordance with prior reports, our study demonstrated that SBP and blood glucose were significantly increased in ACS patients other than CHD patients. Furthermore, SBP, cTn, RBG, and HbA1c were elevated more markedly in the DACS group that the CACS group, while HDL-C was decreased. The miR-92a level was also correlated with changes in HbA1c and SBP. Furthermore, the level of miR-92a was not correlated with blood glucose or lipid level. These findings show that miR-92a can predict ACS events in patients with T2DM and CHD may impair the stability of atherosclerotic plaque.

Except for the traditional risk factors—which include SBP, FIB, cTn, RBG, HbA1c, LDL-C, and HDL-C—the level of miR-92a was significantly associated with the risk of DACS. Further multivariate logistic regression analyses demonstrated that HbA1c, SBP, and miR-92a were independent predictors of ACS in patients with T2DM and CHD. These results held true even after adjusting for group differences. In our study, HbA1c and the average level of blood glucose over weeks or months instead of random blood glucose (RBG) was employed in multivariate logistic regression analysis, which might be because persistent hyperglycemia is a cause of atherosclerosis [28]. Blood lipid levels were also not related to DACS. The average LDL-C of the DACS group was 2.61 mmol/L, which, according to the T2DM management algorithm published by AACE/ACE, is far below the standard of 70 mg/dL (1.8 mmol/L) [29].

Further ROC analyses corroborated that miR-92a separately (cutoff > 4.65) showed 85% sensitivity and 82.50% specificity in predicting ACS. Our forward stepwise binary logistic regression analyses proved that the combination of HbA1c, SBP, and miR-92a had a better predictive value than any individual biomarker taken separately. This combination panel demonstrated 95% sensitivity and 95% specificity in predicting ACS among patients with T2DN and CHD. The progression of diabetes-related atherosclerosis to DACS involves complex mechanisms including hyperglycemia, abnormal lipid metabolism, hemodynamic changes, and vascular inflammatory factors [30]. Currently the possibility of using combinations of biomarkers for the prediction of ACS among patients with T2DM and CHD is of vital importance. Our study shows that a combination of miRNA expression, systolic blood pressure, and blood sugar level may be useful in screening for and identifying ACS among such patients.

### Limitations

There are several limitations to the present study. First, because it is a single-center study with a small sample size, the predictive values should be interpreted with caution. Second, is the absence of pairwise healthy subjects as a control group in whom circulating miR-92a could also be assessed. Because it is known that miRNA-92a plays a role in the pathogenesis of ACS [31], we enrolled only age- and sex-matched patients with chronic stable CHD as a negative control group. Finally, further exploration of the relationship between miR-92a and T2DM-related atherosclerosis and should be undertaken.

## Conclusions

ACS is the primary cause of death in patients with CHD and T2DM. Our study suggests that an elevated level of circulating miR-92a is associated with increased ACS among patients with CHD and T2DM. In our multivariate logistic regression analysis, the level of circulating miR-92a was seen as an independent predictive factor for ACS in patients with CHD and T2DM. Moreover, miR-92a, especially miR-92a combined with HbA1c and elevated SBP, had a better predictive value for ACS in patients with CHD and T2DM. Furthermore, this study also shows that a combination of miRNA expression, elevated systolic blood pressure, and an average level of blood sugar may be helpful in screening patients and identifying those at risk for ACS.

## Additional file

Additional file 1: **Figure S1.** Correlation of miR-92a and hemodynamics parameters. **Figure S2.** Correlation of miR-92a level and blood glucose. **Figure S3.** Correlation of miR-92a level and blood lipid. **Figure S4.** Data from ROC curves. (ZIP 2509 kb)

## Abbreviations

ACS: Acute coronary syndrome
AMI: Acute myocardial infarction
ANOVA: One-way analysis of variance
BMI: Body mass index
BNP: B-type natriuretic peptide
BP: Blood pressure
CHD: Coronary heart disease
cTn: Cardiac troponin
DBP: Diastolic blood pressure
FIB: fibrinogen
HbA1c: Glycated hemoglobin
HDL-C: High-density lipoprotein cholesterol
IQR: Interquartile range
LDL-C: Low-density lipoprotein cholesterol
miRNA: MicroRNA
PPG: Postprandial glucose
RBG: Random blood glucose
ROCs: Receiver operating characteristics
RT-PCR: Real-time polymerase chain reaction
SBP: Systolic blood pressure
T1DM: Type 1 diabetes mellitus
T2DM: Type 2 diabetes mellitus
TC: Total cholesterol
TG: Triglyceride
