# Supplementary figures and images for: Circulating microRNA-92a level predicts acute coronary syndrome in diabetic patients with coronary heart disease
Source: Lipids Health Dis. 2019 Jan 22;18:22. doi: 10.1186/s12944-019-0964-0 (PMC6343303; doi:10.1186/s12944-019-0964-0)

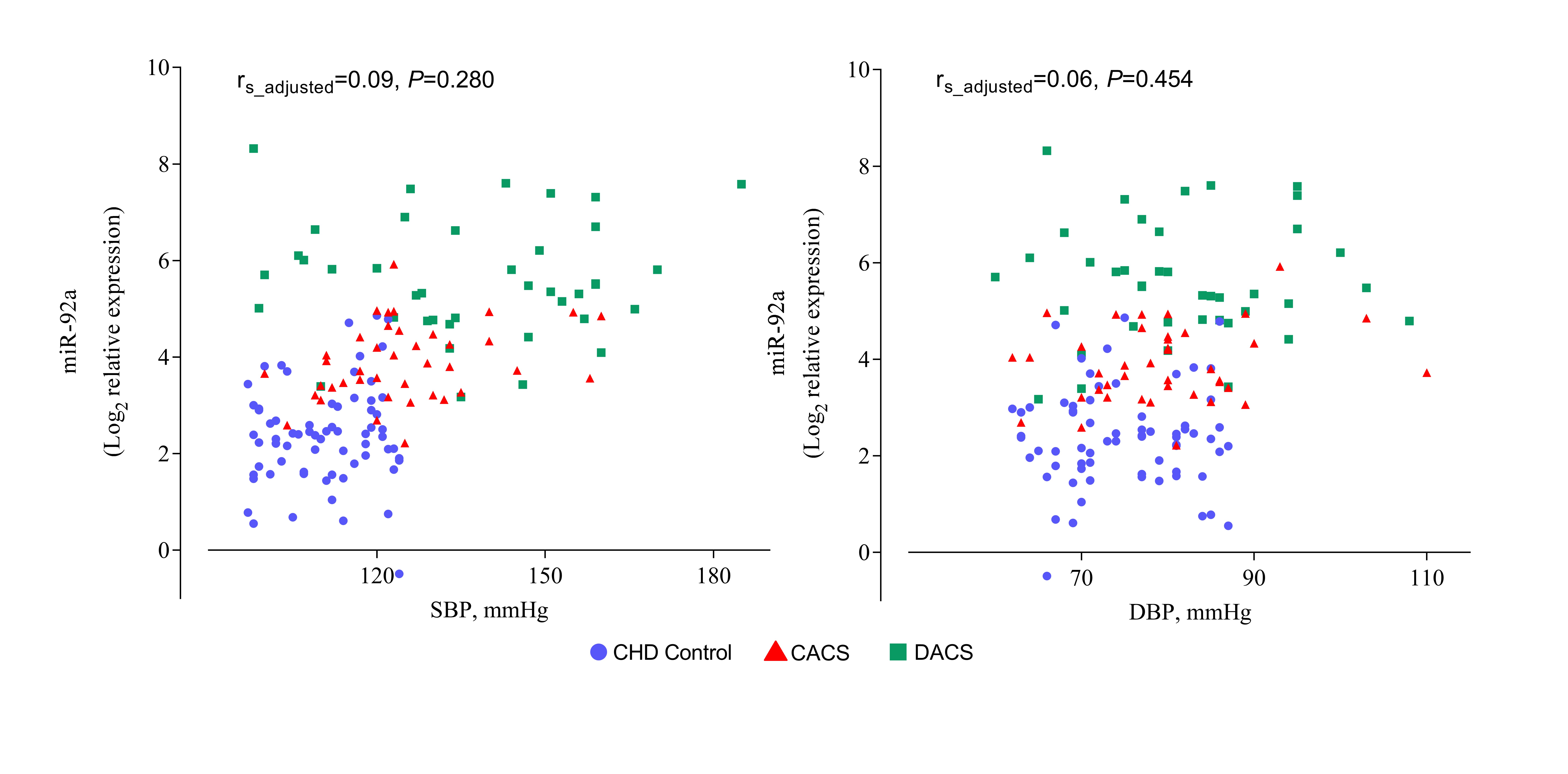

Supplement: Supplementary file 1 — Figure S1. Correlation of miR-92a and hemodynamics parameters. Figure S2. Correlation of miR-92a level and blood glucose. Figure S3. Correlation of miR-92a level and blood lipid. Figure S4. Data from ROC curves. (ZIP 2509 kb) [file 12944_2019_964_MOESM1_ESM.zip › Figure S1.jpg]

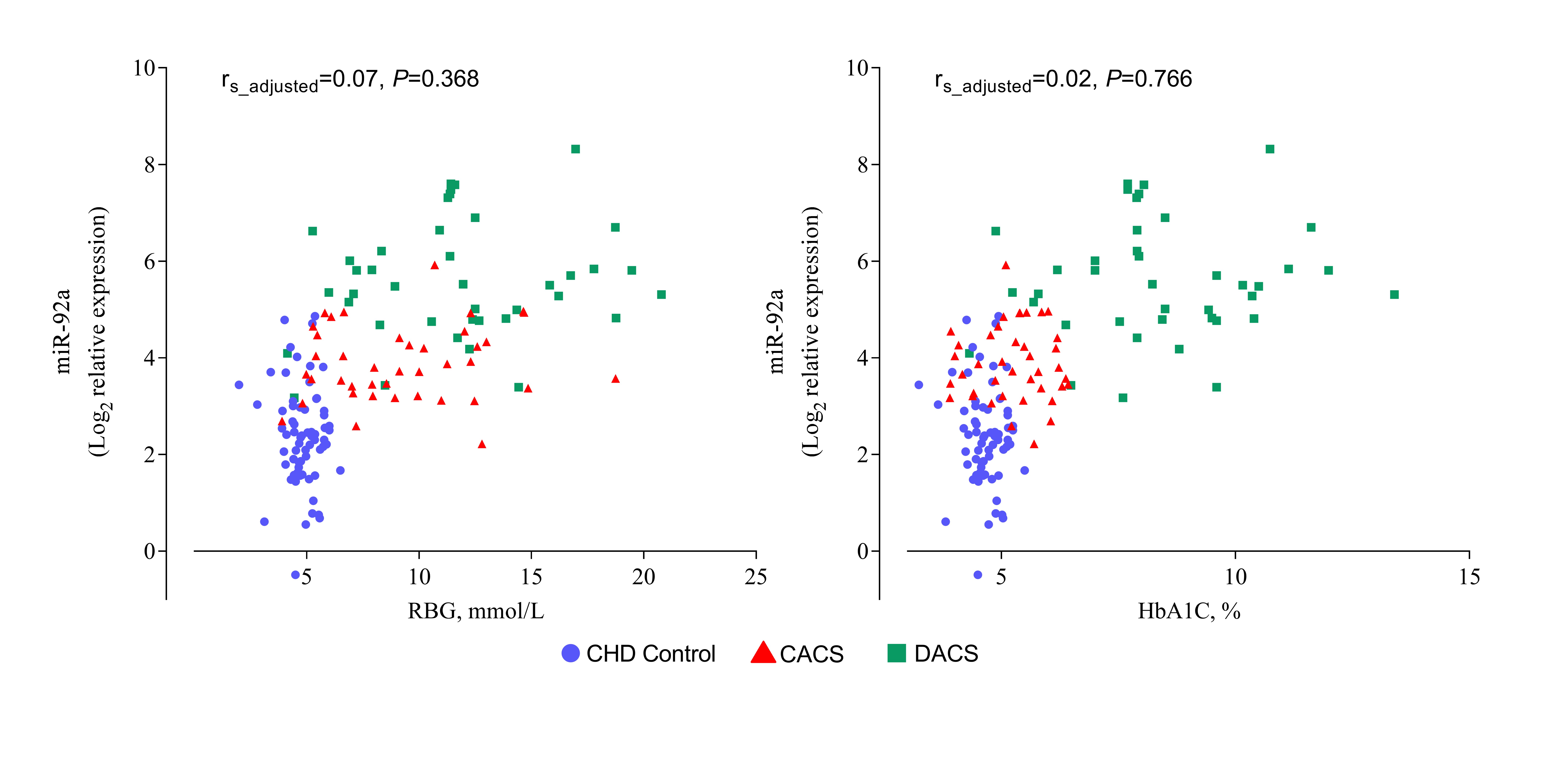

Supplement: Supplementary file 1 — Figure S1. Correlation of miR-92a and hemodynamics parameters. Figure S2. Correlation of miR-92a level and blood glucose. Figure S3. Correlation of miR-92a level and blood lipid. Figure S4. Data from ROC curves. (ZIP 2509 kb) [file 12944_2019_964_MOESM1_ESM.zip › Figure S2.jpg]

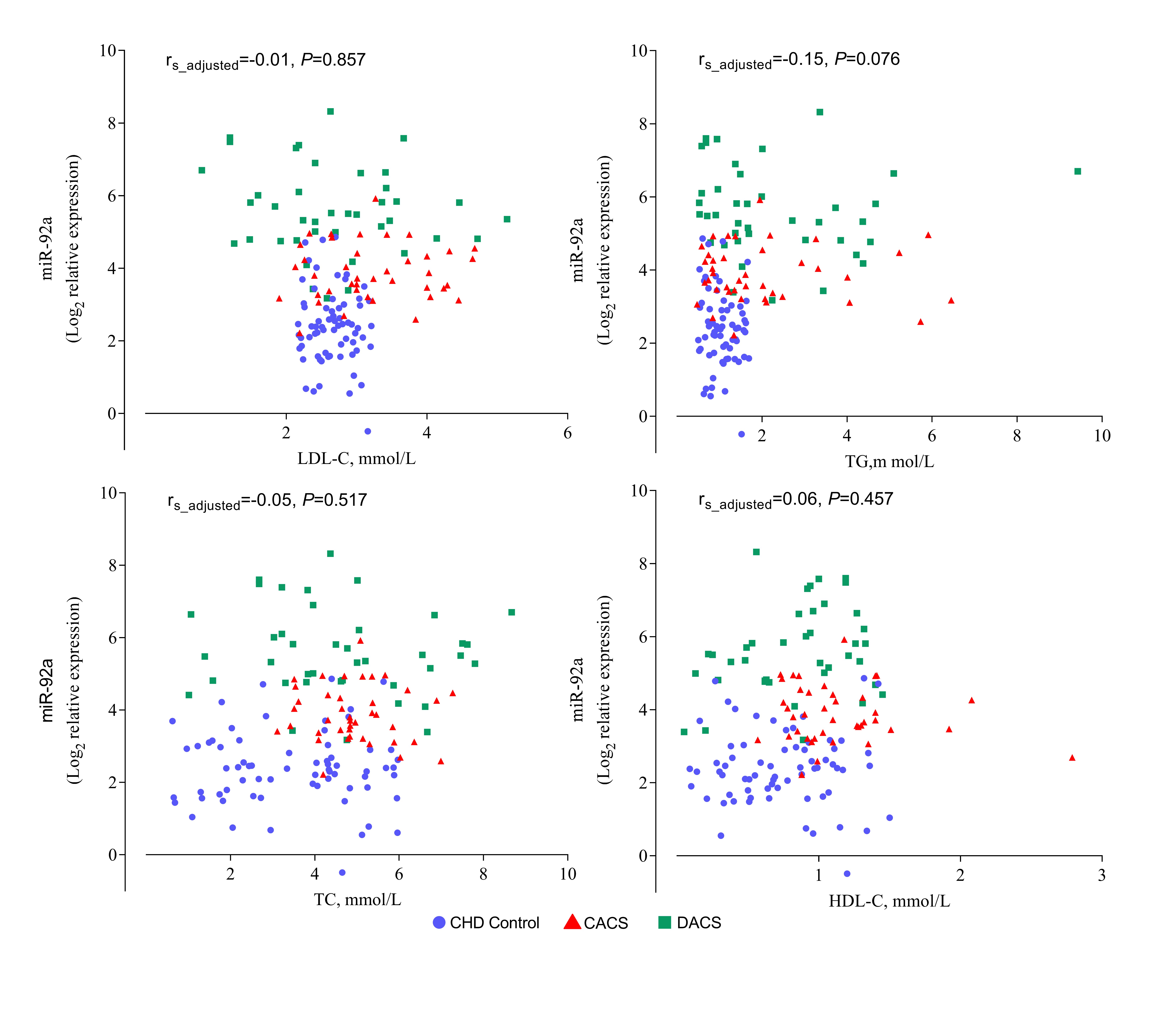

Supplement: Supplementary file 1 — Figure S1. Correlation of miR-92a and hemodynamics parameters. Figure S2. Correlation of miR-92a level and blood glucose. Figure S3. Correlation of miR-92a level and blood lipid. Figure S4. Data from ROC curves. (ZIP 2509 kb) [file 12944_2019_964_MOESM1_ESM.zip › Figure S3.jpg]

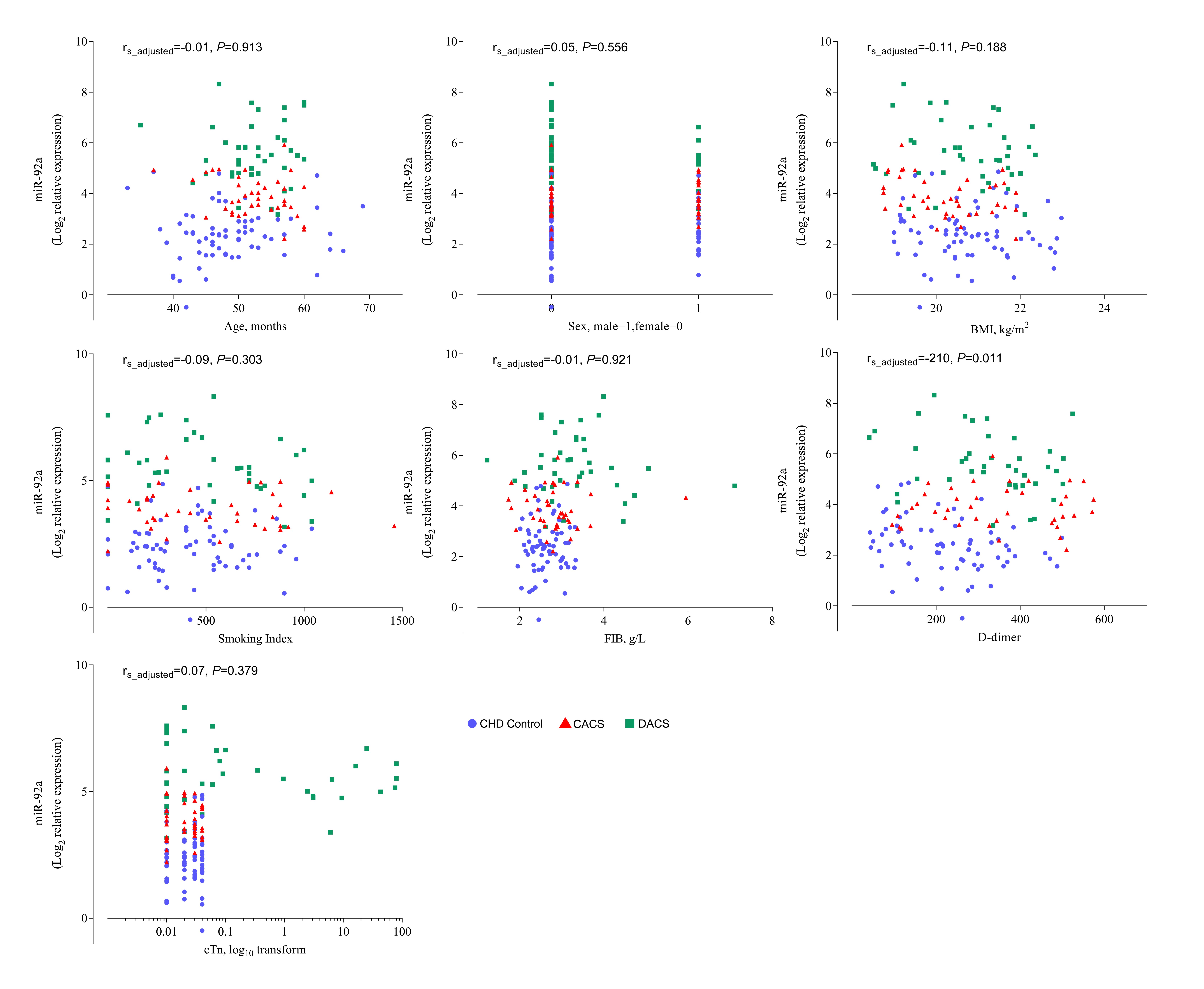

Supplement: Supplementary file 1 — Figure S1. Correlation of miR-92a and hemodynamics parameters. Figure S2. Correlation of miR-92a level and blood glucose. Figure S3. Correlation of miR-92a level and blood lipid. Figure S4. Data from ROC curves. (ZIP 2509 kb) [file 12944_2019_964_MOESM1_ESM.zip › Figure S4.jpg]
